# Comparative study with practical validation of photovoltaic monocrystalline module for single and double diode models

**DOI:** 10.1038/s41598-021-98593-6

**Published:** 2021-09-27

**Authors:** Salam J. Yaqoob, Ameer L. Saleh, Saad Motahhir, Ephraim B. Agyekum, Anand Nayyar, Basit Qureshi

**Affiliations:** 1 Authority of the Popular Crowd, Baghdad, Iraq; 2 College of Engineering, Misan University, Amarah, Iraq; 3 Ecole National des sciences appliquées, Université Sidi Med Ben Abdellah, Fes, Morocco; 4 Department of Nuclear and Renewable Energy, Ural Federal University, 19 Mira Street, Ekaterinburg, Russia; 5 Graduate School, Duy Tan University, Da Nang, Vietnam; 6 College of Computer & Info Sc., Prince Sultan University, Riyadh, Saudi Arabia

**Keywords:** Energy science and technology, Engineering

## Abstract

A photovoltaic (PV) module is an equipment that converts solar energy to electrical energy. A mathematical model should be presented to show the behavior of this device. The well-known single-diode and double-diode models are utilized to demonstrate the electrical behavior of the PV module. “Matlab/Simulink” is used to model and simulate the PV models because it is considered a major software for modeling, analyzing, and solving dynamic system real problems. In this work, a new modeling method based on the “Multiplexer and Functions blocks” in the "Matlab/Simulink Library" is presented. The mathematical analysis of single and double diodes is conducted on the basis of their equivalent circuits with simple modification. The corresponding equations are built in Matlab by using the proposed method. The unknown internal parameters of the PV panel circuit are extracted by using the PV array tool in Simulink, which is a simple method to obtain the PV parameters at certain weather conditions. Double-diode model results are compared with the single-diode model under various irradiances and temperatures to verify the performance and accuracy of the proposed method. The proposed method shows good agreement in terms of the *I*–*V* and *P*–*V* characteristics. A monocrystalline NST-120 W PV module is used to validate the proposed method. This module is connected to a variable load and tested for one summer day. The experimental voltage, current, and power are obtained under various irradiances and temperatures, and the *I*–*V* and *P*–*V* characteristics are obtained.

## Introduction

Renewable energy is the best source of electricity because it is free, clean, and highly abundant. Renewable energy gained by photovoltaic (PV) modules is the most common source^1^. A PV cell is a device that converts solar energy to electrical energy, and has a behavior similar to a P–N junction. The output voltage of these cells should be raised by connecting them in series to form a PV module. These cells are also connected in parallel to obtain a large output current. This connection is integrated to form a large PV system with a higher power, which is called an array^2^. The PV module’s output power primarily depends on irradiation and cell temperature. The voltage drops, and the current rises slightly whenever the temperature increases. Thus, the PV system efficiency is decreased. Different types of PV cell technologies are currently made on the market, depending on the commercial maturity and manufacturing materials. These types can be summarized into three major technologies, namely, polycrystalline, monocrystalline, and thin-film technologies^3,4^. Polycrystalline technology is relatively inefficient due to the arrangement of crystals, which is random, and the cell’s color is slightly bluer, reflecting more of the sunlight. Monocrystalline technology is extremely efficient. This technology absorbs extremely high sunlight radiation because it has a uniform black color. The efficiency of this technology is more than that of polycrystalline. The manufacturing cost of polycrystalline technology is lower than pure silicon wafers. Thin-film technology is more efficient than other technologies. This technology is made of a thin layer of amorphous film that elicits more energy from the available sunlight. These technologies of the PV cell are combined with the same point in the physical behavior of a diode P–N junction^5–8^.

The main drawback of the PV module is the lower efficiency. Thus, a maximum power point tracking (MPPT) was proposed^9–12^ to elicit the maximum power and enhance the efficiency of the PV module. The modeling representation of the PV panel is extremely important to show the PV characteristics under different weather conditions. Many researchers have developed single diode and double-diode PV models, and a large body of literature is found on this topic^13,14^. A simple PV model was explored by using a single-diode model with four parameters^15,16^. The electrical PV module circuit was built by using a photocurrent source, a diode, a series resistor, and an ideality diode constant. The accuracy of this model is lower compared with that proposed in^17^ that added a shunt resistor to achieve a new model with five parameters.

Several studies have developed PV panel models to solve this issue. In^18–22^, a double-diode PV model is presented to increase the accuracy of the panel model performance. In these studies, a photocurrent source, two diodes, a series resistor, a shunt resistor, and an ideality diode constant are used to form a PV model circuit. A more complex simulation model is utilized to represent the PV panel equivalent circuit and extract the PV characteristics of *I*–*V* and *P*–*V* curves under different irradiances and temperatures. The authors in^23^ presented a new method to determine the PV parameters of a double-diode circuit to enhance its efficiency and accuracy. The drawback of this method is the complex computational process to obtain circuit parameters, such as ideality constant, series, and shunt resistances. Therefore, the researchers in^24–27^ reduced the complexity of the PV circuit by using a five-parameter model. In^24–27^, this model is insufficient to depict a real PV circuit although the accuracy of the *I*–*V* and *P–V* characteristics are not mentioned. A more accurate PV circuit model based on two-diode model is proposed in^28^. The seven parameters of this model are computed by using a hybrid method consists of numerical and analytical methods. Single- and double-diode models are proposed in^29^. They are simulated to determine their difference under various irradiances and temperatures. In this work, an optimization method is used to compute the circuit parameters. Although these studies have achieved good results in terms of PV performance, they use a complicated and difficult modeling method.

Reference^30^ conducted implicit modeling of two-diode model for PV array configuration. Each subpanel is considered by the implicit expression derived to represent a series–parallel array on the basis of double-diode cell circuit. The resultant system equations are solved by using trust-region dogleg method to extract the PV circuit parameters used in every array. This method achieves satisfactory results are achieved in terms of *I–V* and *P–V* characteristics for different weather conditions. The researchers in^31,32^ proposed a new method to model and extract the parameters of PV cell or module by using flower pollination method. A double-diode PV circuit is used to obtain a high accuracy PV model. The results obtained are compared with other results that used optimization techniques, such as artificial bee swarm optimization, pattern search, and harmony search, to prove the effectiveness of the proposed method. However, most of these studies require a high computational cost, making it extremely difficult for users. The authors in^33^ presented a simple representation for PV circuit model. The proposed method is based on the stepwise simplification of the total current equation of the PV equivalent circuit for one and two diodes. The proposed method is investigated by using LTspice simulator tool. The authors in^34–36^ developed a double-diode model by adding another parallel diode in PV equivalent circuit. This model increases the power losses due to the leakage current in the third diode and reduces the total output power. Thus, the accuracy of the *I*–*V* and *P*–*V* characteristics of the PV panel is affected, especially when the PV panel works under lower irradiance levels.

In this study, a simple and new method for modeling the double-diode PV model in Matlab is presented. This method is built on the basis of the simple mathematical equations of reverse saturation diode currents. The double-diode PV parameters are extracted with a simple PV array tool presented in a new version of Simulink (2016), which is released by Mathworks. The simulation results obtained by this method are compared with that obtained by the single-diode model at standard test conditions (STCs). The *I*–*V* and *P*–*V* curves of the two models are achieved and validated with a 120 W-PV module to test the performance under different weather conditions.

The rest of this paper is organized as follows: Sect. 2 presents the PV cell models. Section 3 introduces the modeling of PV panel. Section 4 highlights the hardware implementation adopted in this research. Section 5 discusses the results. Section 6 presents the conclusion.

## PV cell models

### Single-diode model

**Figure 1:**
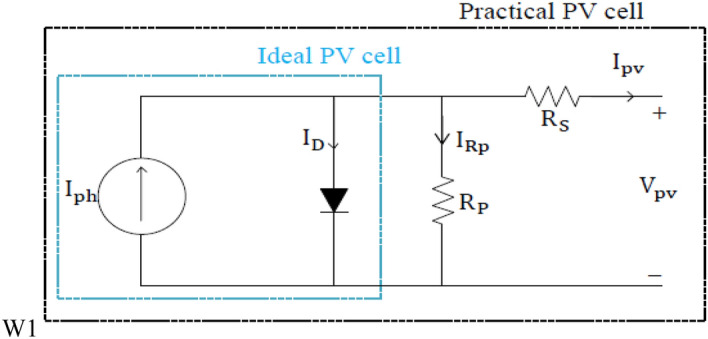
Single-diode model of the PV cell.

As shown in Fig. 1, the PV cell model is a single-diode model because it is built on the assumption that the recombination failure in the depletion area is negligible. The loss of the P–N junction’s depletion area is important, which is invisible in the single-diode configuration. The basic equation of semiconductor diode theory represents the characteristics of an ideal PV cell as follows^9,13^:

1 $${\mathrm{I}}_{\mathrm{pv}}={\mathrm{I}}_{\mathrm{ph}}-{\mathrm{I}}_{0}\left[\mathrm{exp}\left(\frac{\mathrm{q }{\mathrm{V}}_{\mathrm{pv}}}{\mathrm{\alpha K T}}\right)-1\right]$$

Equation (1) does not symbolize the real behavior of the PV cell. For this reason, a small milliohm of a series resistor with a high value of a parallel resistor is inserted into the equivalent circuit of the PV cell circuit. The PV cell current can be expressed as^17,18^

2 $${\mathrm{I}}_{\mathrm{pv}}={\mathrm{I}}_{\mathrm{ph}}-{\mathrm{I}}_{0}\left[\mathrm{exp}\left(\frac{\mathrm{q }{\mathrm{V}}_{\mathrm{pv}}}{\mathrm{\alpha }{\mathrm{V}}_{\mathrm{T}}}\right)-1\right]-\frac{{\mathrm{V}}_{\mathrm{pv}}+{\mathrm{R}}_{\mathrm{s }}{\mathrm{I}}_{\mathrm{pv}}}{{\mathrm{R}}_{\mathrm{P}}}$$

The source of photocurrent is linearly proportional to irradiance and is influenced by temperature, as shown in Eq. (3). ^20^.

3 $${\mathrm{I}}_{\mathrm{ph}}=\left({\mathrm{I}}_{\mathrm{phn}}+{\mathrm{K}}_{\mathrm{i}}\Delta \mathrm{T}\right)\frac{\mathrm{G}}{{\mathrm{G}}_{\mathrm{n}}}$$

where $\Delta \mathrm{T}=\mathrm{T}-{\mathrm{T}}_{\mathrm{n}}$ (T_n _= 25 °C), $\mathrm{G}$ is the incident of irradiation on the module, and ${\mathrm{G}}_{\mathrm{n}}$(1000 W/m^2^) at $\mathrm{STC}$. The diode saturation current can be written as^16^

4 $${\mathrm{I}}_{0}={\mathrm{I}}_{0\mathrm{n}}{\left(\frac{{\mathrm{T}}_{\mathrm{n}}}{\mathrm{T}}\right)}^{3}\mathrm{exp}\left[\frac{{\mathrm{qE}}_{\mathrm{g}}}{\mathrm{\alpha K}}\left(\frac{1}{{\mathrm{T}}_{\mathrm{n}}}-\frac{1}{\mathrm{T}}\right)\right]$$

A modified equation that describes the current in the saturation case is shown below^6^.

5 $${\mathrm{I}}_{0}=\frac{\left({\mathrm{I}}_{\mathrm{scn}}+{\mathrm{K}}_{\mathrm{i}}\Delta \mathrm{T}\right)}{\mathrm{exp}\left[\frac{\left({\mathrm{V}}_{\mathrm{ocn}}+{\mathrm{K}}_{\mathrm{v}} \Delta \mathrm{T}\right)}{\mathrm{\alpha }{\mathrm{V}}_{\mathrm{T}}}\right]-1}$$

This modification aims to make the open-circuit voltage of the model match that of the experiment. The saturation current is influenced by the variation of the temperate. This modification facilitates the model and eliminates the model’s error on open-circuit voltages for the regions of the *I*–*V* characteristic. The terms of the previous equations are presented as:

- ${\mathrm{I}}_{\mathrm{pv}}$ is the PV output current.
- ${\mathrm{V}}_{\mathrm{pv}}$ is the output voltage of the PV module.
- ${\mathrm{I}}_{\mathrm{D}}$ is the diode current.
- ${\mathrm{I}}_{\mathrm{ph}}$ is the photocurrent source.
- ${\mathrm{I}}_{\mathrm{o}}$ is the saturation diode current.
- ${\mathrm{I}}_{\mathrm{on}}$ represents the saturation current at STC condition.
- ${\mathrm{I}}_{\mathrm{phn}}$ is the photocurrent at STC condition.
- $\mathrm{T}$ is the ambient temperature
- ${\mathrm{V}}_{\mathrm{T}}\left(={\mathrm{N}}_{\mathrm{S}}\mathrm{K T}/\mathrm{q}\right)$ is the thermal voltage.
- ${\mathrm{N}}_{\mathrm{S}}$ is the number of cells per module.
- ${\mathrm{R}}_{\mathrm{P}}$ and ${\mathrm{R}}_{\mathrm{S}}$ are the parallel and series resistances, respectively.
- ${\mathrm{K}}_{\mathrm{i}}$ is the thermal coefficient at ${\mathrm{I}}_{\mathrm{sc}}$.
- $\mathrm{\alpha }$ is the diode ideality factor.
- ${\mathrm{K}}_{\mathrm{V}}$ is the thermal coefficient at ${\mathrm{V}}_{\mathrm{oc}}$.
- ${\mathrm{E}}_{\mathrm{g}}$ is the band gap energy.
- $\mathrm{q}$ is the electron charge (1.602 × 10^−19^ °C).
- $\mathrm{K}$ is the Boltzmann constant ($1.3806\times {10}^{-23}\mathrm{J}/\mathrm{K}).$

### Double-diode model

**Figure 2:**
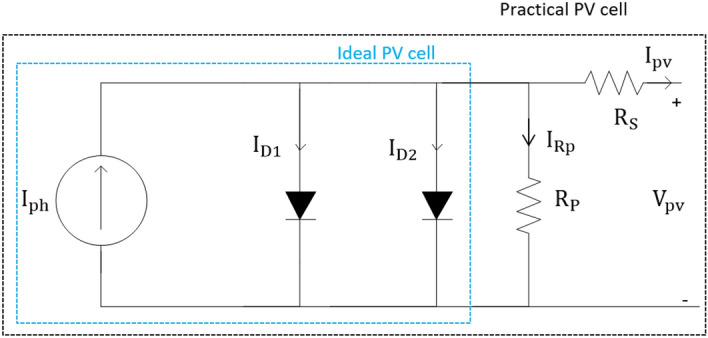
Double-diode PV model circuit.

The lack of recombination that is ignored in the single**–**diode model causes the inaccuracy of the PV model parameters. Therefore, the double-diode model is chosen to represent the physical form of the PV cell, as shown in Fig. 2. A precise model is achieved by considering recombination loss. The diffusion current is focused in the p–n junction material by using one of the double diodes, and the other is added to account for recombination loss^20,21^. Hence, the PV module output current can be defined as:

6 $${\mathrm{I}}_{\mathrm{pv}}={\mathrm{I}}_{\mathrm{ph}}-{\mathrm{I}}_{\mathrm{D}1}-{\mathrm{I}}_{\mathrm{D}2}-\left[\frac{{\mathrm{V}}_{\mathrm{pv}}+{\mathrm{I}}_{\mathrm{pv}}{\mathrm{R}}_{\mathrm{s}}}{{\mathrm{R}}_{\mathrm{P}}}\right]$$

7 $${\mathrm{I}}_{\mathrm{D}1}={\mathrm{I}}_{01}\left[\mathrm{exp}\left(\frac{{\mathrm{V}}_{\mathrm{pv}}+{\mathrm{I}}_{\mathrm{pv}}{\mathrm{R}}_{\mathrm{s}}}{{\mathrm{\alpha }}_{1}{\mathrm{V}}_{\mathrm{T}1}}\right)-1\right]$$

8 $${\mathrm{I}}_{\mathrm{D}2}={\mathrm{I}}_{02}\left[\mathrm{exp}\left(\frac{{\mathrm{V}}_{\mathrm{pv}}+{{\mathrm{I}}_{\mathrm{pv}}\mathrm{R}}_{\mathrm{s}}}{{\mathrm{\alpha }}_{2}{\mathrm{V}}_{\mathrm{T}2}}\right)-1\right]$$

As mentioned previously, a more accurate model can be realized by using a two-diode model. Seven parameters, namely, ${I}_{\mathrm{ph}}$, ${I}_{01}$, ${I}_{02}$,${\alpha }_{1}$, ${\alpha }_{2}$,${R}_{s}$, and ${R}_{\mathrm{P}}$ must be calculated. Some studies have used iteration methods to calculate the values of saturation currents for double-diode models (${I}_{01}$ and ${I}_{02}$)^7,8^. ${I}_{01}$ is approximately 3–7 orders larger than that of ${I}_{02}$. ${\alpha }_{1}$ and ${\alpha }_{2}$ are taken as 1 and 2, respectively, to simplify the calculation. Thus, the saturation currents can be expressed as^14,19^

9 $${\mathrm{I}}_{01}=\frac{\left({{\mathrm{I}}_{\mathrm{scn}}}_{ }+{\mathrm{K}}_{\mathrm{i}} \Delta \mathrm{T}\right)}{\mathrm{exp}\left[\frac{\left({\mathrm{V}}_{\mathrm{ocn}}+{\mathrm{K}}_{\mathrm{v}} \Delta \mathrm{T}\right)}{{\mathrm{\alpha }}_{1}{\mathrm{V}}_{\mathrm{T}1}}\right]-1}$$

10 $${\mathrm{I}}_{02}=\frac{\left({{\mathrm{I}}_{\mathrm{scn}}}_{ }+{\mathrm{K}}_{\mathrm{i}} \Delta \mathrm{T}\right)}{\mathrm{exp}\left[\frac{\left({\mathrm{V}}_{\mathrm{ocn}}+{\mathrm{K}}_{\mathrm{v}} \Delta \mathrm{T}\right)}{{\mathrm{\alpha }}_{2}{\mathrm{V}}_{\mathrm{T}2}}\right]-1}$$

When the terminals of the PV panel are tested under open-circuit operation, the ambient temperature should be considered, which is affected on the *I*–*V* and *P*–*V* characteristics during different temperature conditions. Thus, the output voltage can be expressed as^3^

11 $${\mathrm{V}}_{\mathrm{oc}}={\mathrm{V}}_{\mathrm{ocn}}+{\mathrm{K}}_{\mathrm{V}}{ \Delta }_{\mathrm{T}}$$

### Extracted PV parameters

**Figure 3:**
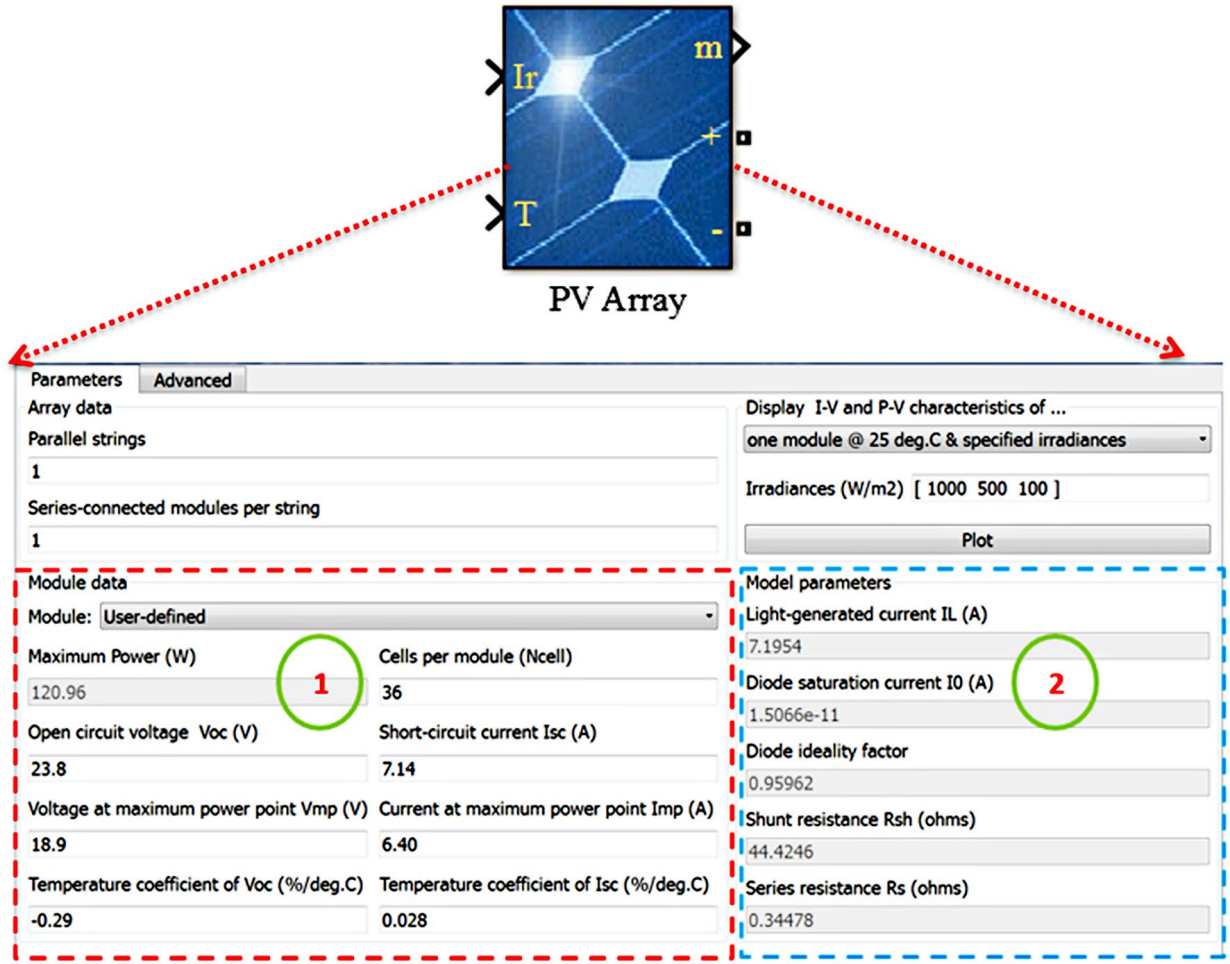
Extracted parameters of the NST-120 panel.

The PV panel datasheet does not contain some important parameters, such as ${R}_{\mathrm{s}}$, ${R}_{\mathrm{P}}$, ${I}_{\mathrm{o}}$, and $\alpha$, that are used in the modeling process. Therefore, these parameters are extremely important in modeling PV panels. They are used to represent the real circuit of the PV Panel. However, several methods are presented and reviewed to determine these parameters. This study utilized a simple and sufficient method of PV array Matlab/tool to compute these parameters. We use this tool provided in Matlab 2016 to set the datasheet parameters by simply clicking on the tool window, and the extracted parameters are obtained, as shown in Fig. 3. Block (1) refers to the datasheet parameters, and block (2) presents the extracted PV parameters. Table 1 demonstrates the datasheet parameters of the NST-120 PV panel that is utilized in this study. The extracted parameters are shown in Table 2.

**Table 1:** Parameters of the NST-120 PV panel at STC from the datasheet.

Parameter	Value	Unit	
${P}_{\mathrm{mp}}$	120	W	
${V}_{\mathrm{mp}}$	18.9	V	
${I}_{\mathrm{mp}}$	6.40	A	
${V}_{\mathrm{oc}}$	23.8	V	
${I}_{\mathrm{sc}}$	7.14	A	
${K}_{\mathrm{i}}$	0.028	%/°C	
${K}_{\mathrm{V}}$	− 0.29	%/°C	
${\mathrm{N}}_{\mathrm{S}}$	36	–

**Table 2:** Extracted parameters of the NST-120 PV panel by using PV array tool.

Quantity	Value	Unit
${{\varvec{R}}}_{\mathbf{S}}$	0.344	$\Omega$
${{\varvec{R}}}_{\mathbf{P}}$	44.42	$\Omega$
${{\varvec{I}}}_{\mathbf{o}}$	$1.5066\times {10}^{-11}$	A
$\boldsymbol{\alpha }$	0.9596	–

## Modeling of PV panel

**Figure 4:**
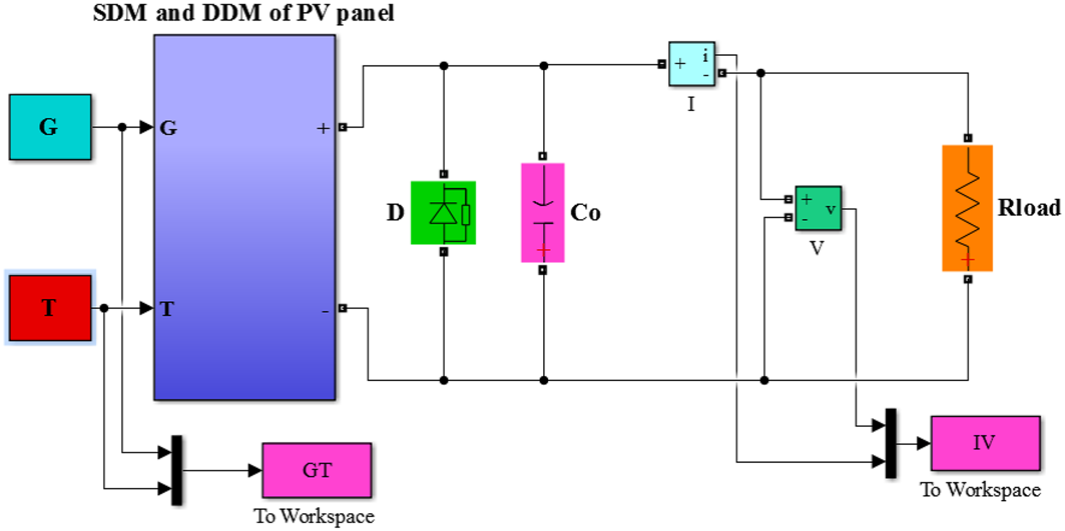
Simulink/Matlab block diagram of the NST-120 PV panel.

**Figure 5:**
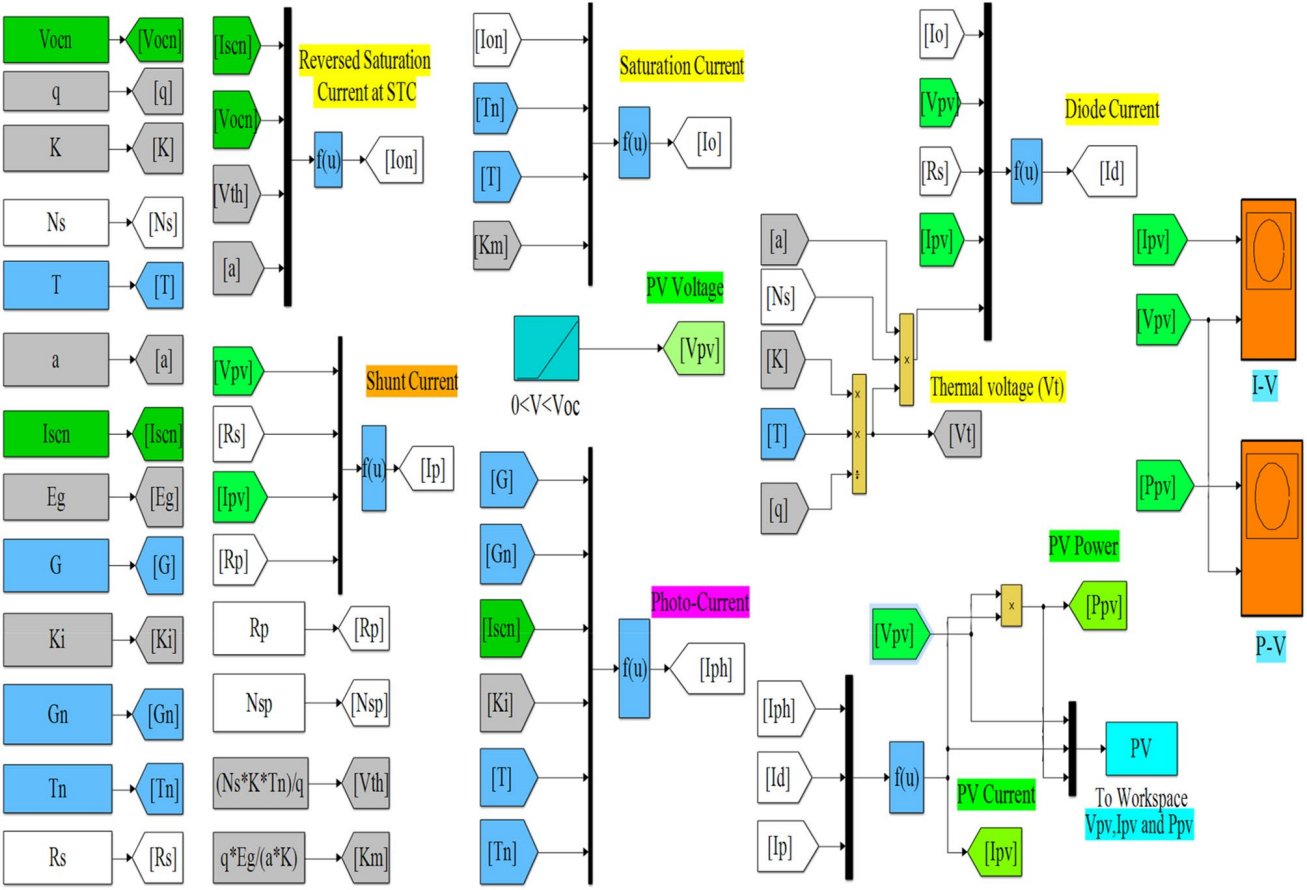
Single-diode model in Matlab.

**Figure 6:**
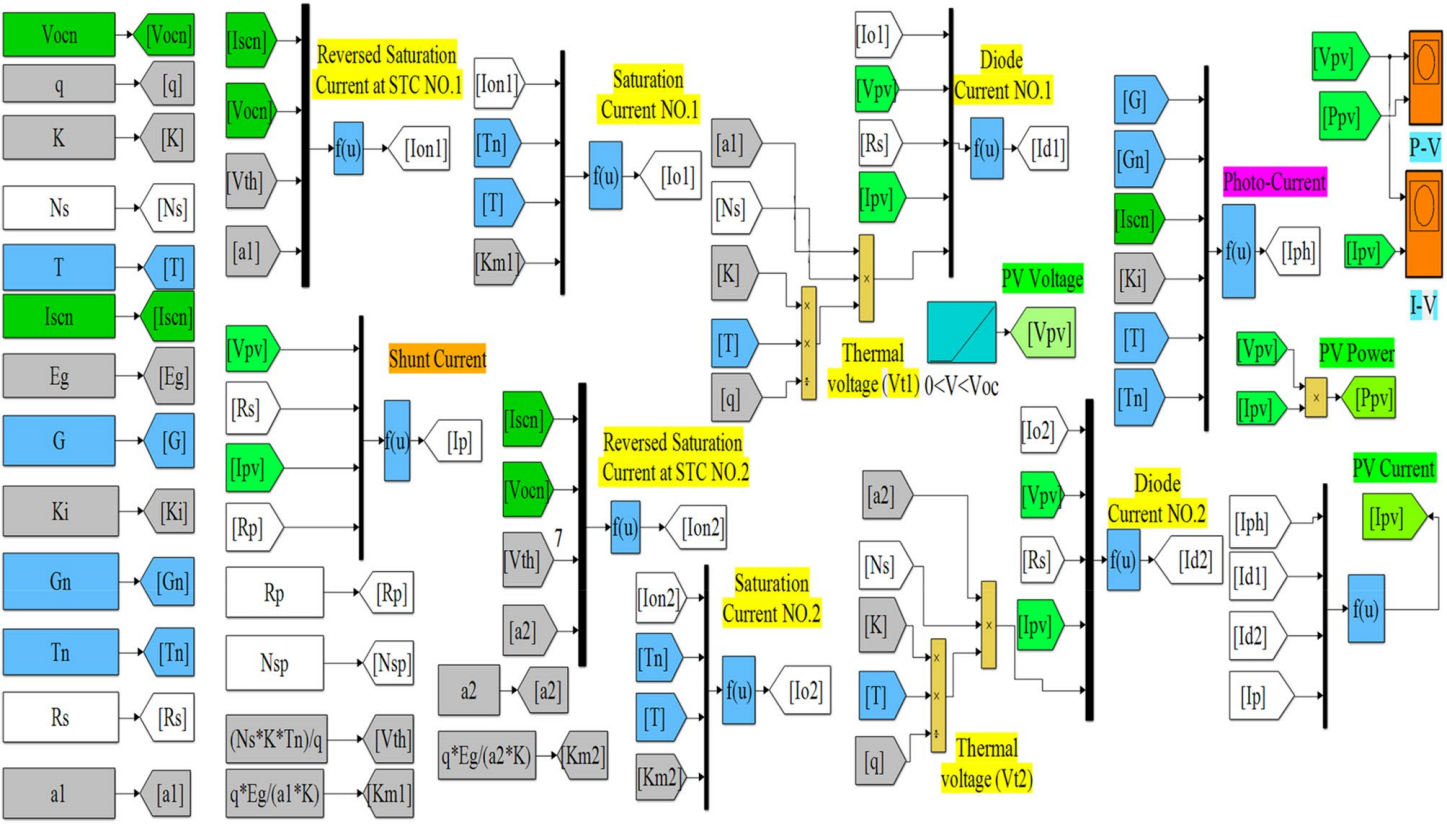
Double-diode model in Matlab.

The datasheet and extracted parameters of the NST panel are used to simulate the single- and double-diode models for representing the *P*–*V* and *I*–*V* panel characteristics, as shown in Fig. 4. A simple simulation method of Matlab is used to obtain the PV graphs. This method is based on two utilized tools to represent the equations given in Sect. (2.1) of the single-diode model and the equations given in Sect. (2.2). These models are shown in Figs. 5 and 6. The two main tools utilized in this work are as follows:

- Multiplexer block (Mux): this block is utilized from “library Simulink/Signal Routing”.
- Function block (Fcn): this block is utilized from “library Simulink/User-Defined Functions”.

## Hardware implementation

**Figure 7:**
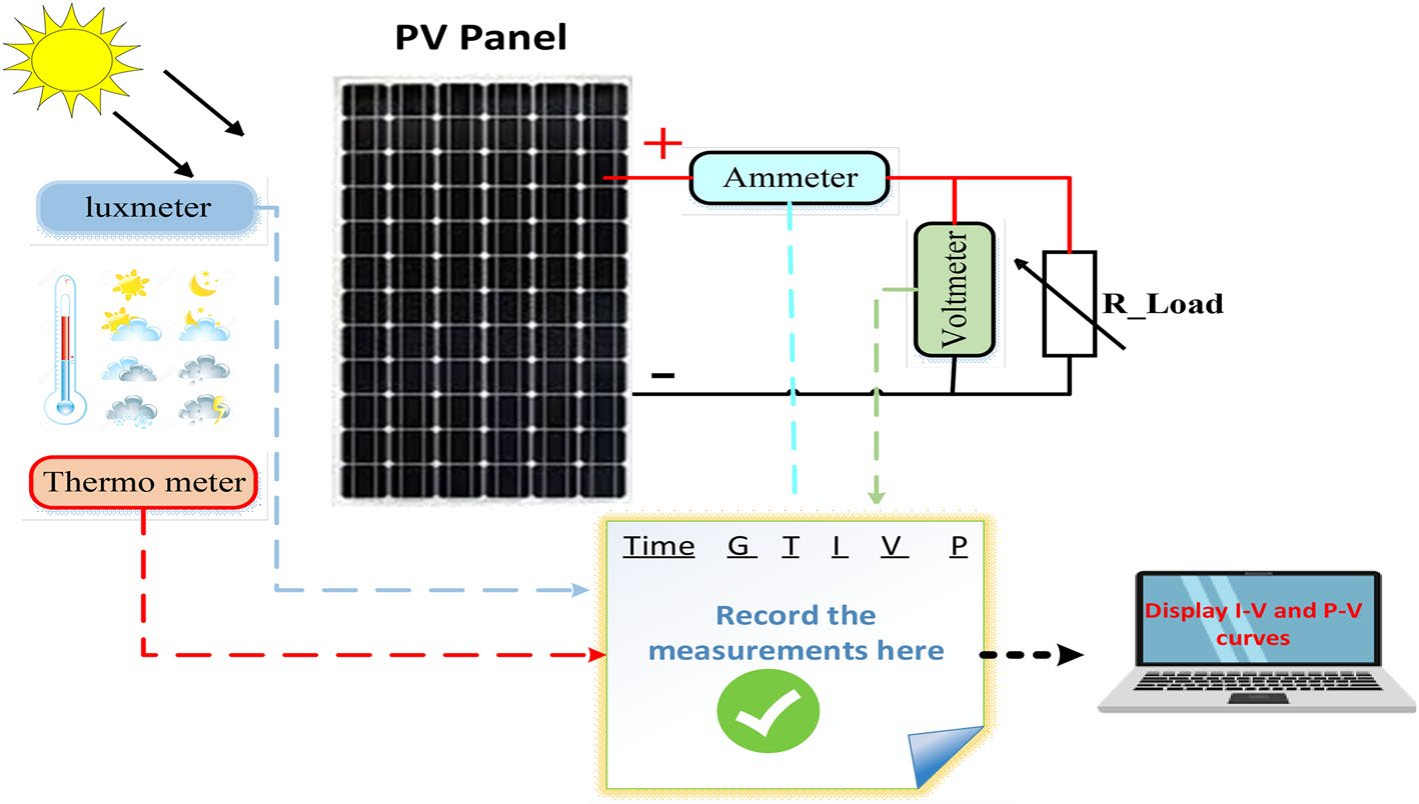
Proposed PV system components.

The NST panel is integrated with practical measurement devices to validate its performance under different values of irradiance and temperature, as shown in Fig. 7. This system consists of a lux meter to measure solar irradiance, a thermometer to sense temperature, ammeter, and voltmeter. A lux meter is used to measure irradiance practically and show the influence of solar irradiance values on the PV panel performance. This device shows the solar irradiance in Lux unit (1–50,000 Lux), where 1 Lux equals 0.79 W/m^2^. The ambient temperature in the experiment is measured by using a thermometer. This device offers an additional feature of humidity measurement, which is a low-cost simplicity for the user. The PV panel is connected to the variable resistive load and the corresponding voltage, and the current is extracted during a sunny day, as shown in Sect. 5.2.

## Results and discussion

### Simulation results

**Figure 8:**
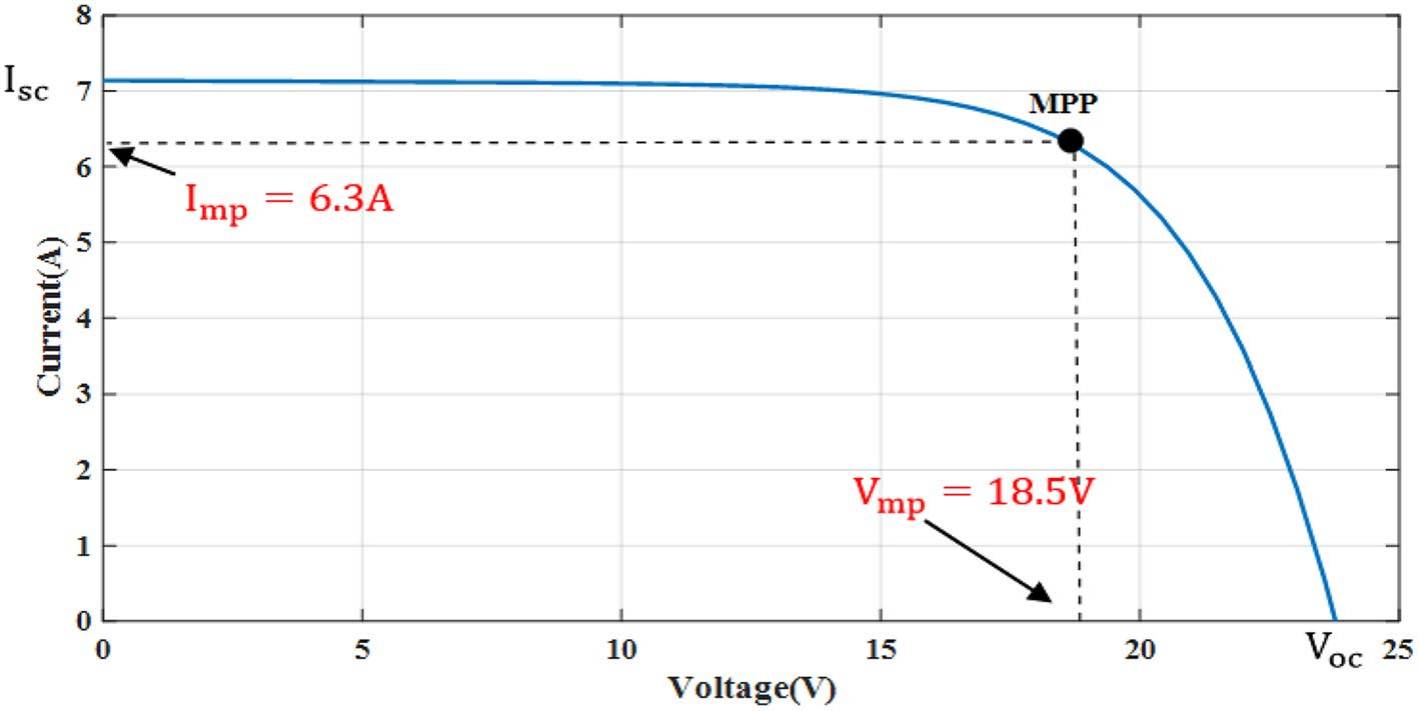
*I*–*V* graph of single-diode model at STC.

**Figure 9:**
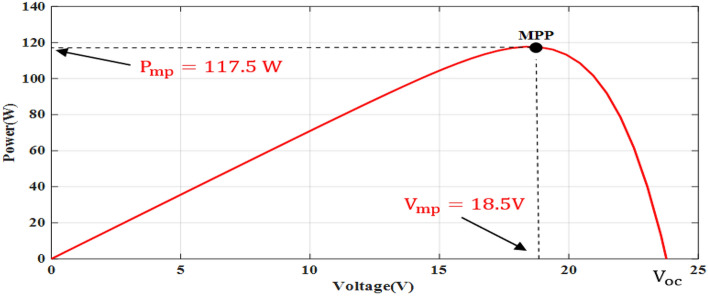
*P*–*V* graph of single-diode model at STC.

**Figure 10:**
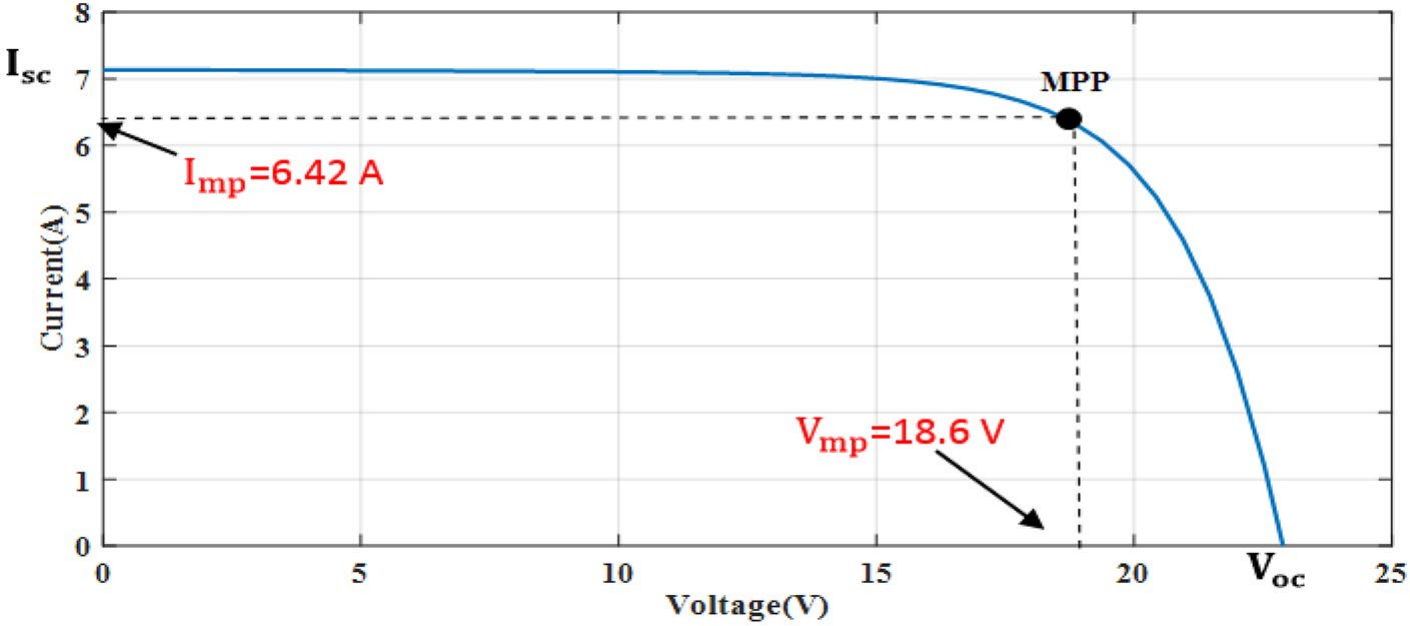
*I*–*V* graph of double-diode model at STC.

**Figure 11:**
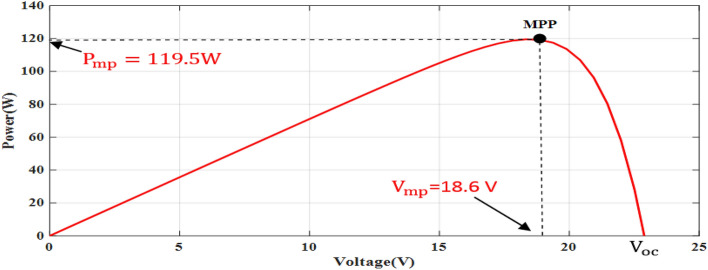
*P*–*V* graph of double-diode PV model at STC.

**Figure 12:**
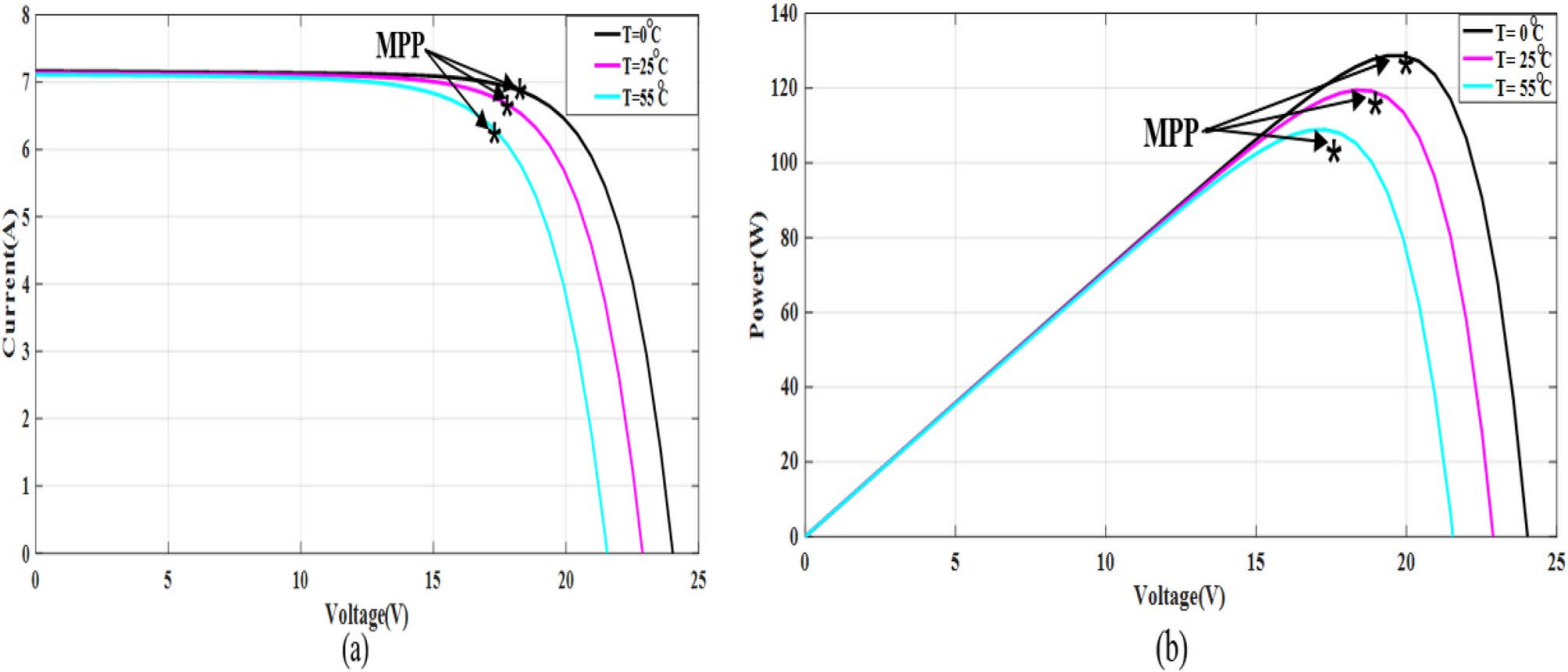
*I*–*V* and *P*–*V* graphs of double-diode model at different values of temperature and fixed irradiation, $G=1000$ W/m^2^ (**a**) *I*–*V* graph and (**b**) *P*–*V* graph.

**Figure 13:**
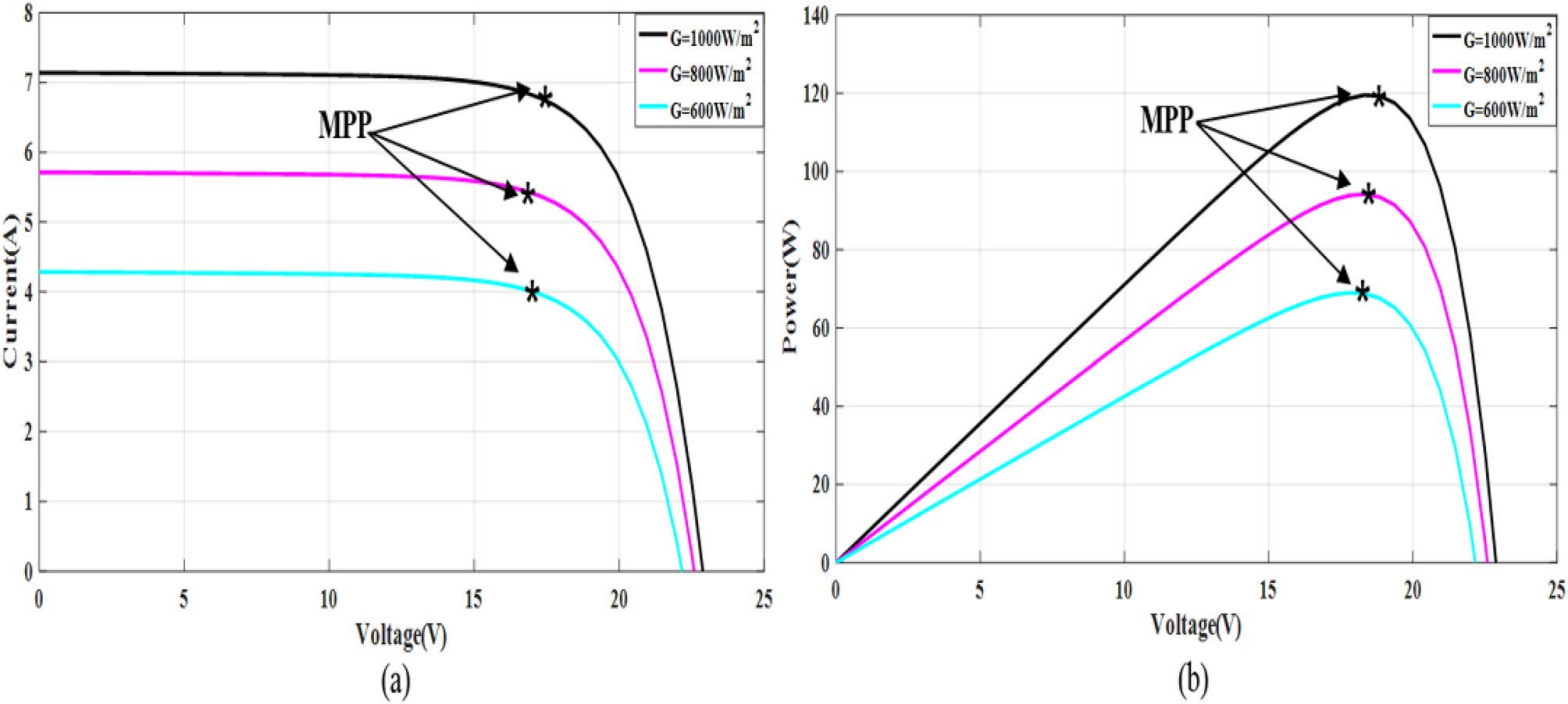
*I*–*V* and *P*–*V* graphs of double-diode model under different values of irradiation and constant temperature, *T* = 25 °C (**a**) *I*–*V* graph and (**b**) *P*–*V* graph.

The simple Matlab method combined from Fcn and Mux is used to verify the proposed method. The single-diode model is represented, and its simulation results are achieved to show the real PV module characteristics under different solar insulations and ambient temperatures. Figures 8 and 9 depict the *I*–*V* and *P–V* graphs for the single-diode model at STC of $T$ = 25 °C and $G$ = 1000 W/m^2^. The double-diode model results are shown in Figs. 10 and 11, showing the *I*–*V* and *P*–*V* graphs at STC. The double-diode model presents higher accuracy in point short-circuit current region, MPP region, and open-circuit voltage region. The double-diode model is simulated under various irradiances and ambient temperatures, as shown in Figs. 12 and 13. When the irradiation level is high, the open-circuit voltage is increased logarithmically, and the current is increased linearly in accordance with Eq. (3) in Sect. 2.1.If the ambient temperature is high, the open-circuit voltage becomes low due to the sign of ${K}_{\mathrm{v}}$, which is negative, as presented in Eq. (11) in Sect. 2.2. The PV current increases slightly in accordance with ${K}_{\mathrm{i}}$ constant, which is positive and extremely small. In this simulation, the value of the optimal resistance load is used on the basis of the MPP point at STC, ${R}_{\mathrm{opt}}=\frac{{V}_{\mathrm{mp}}}{{I}_{\mathrm{mp}}}$.

### Experimental results

**Figure 14:**
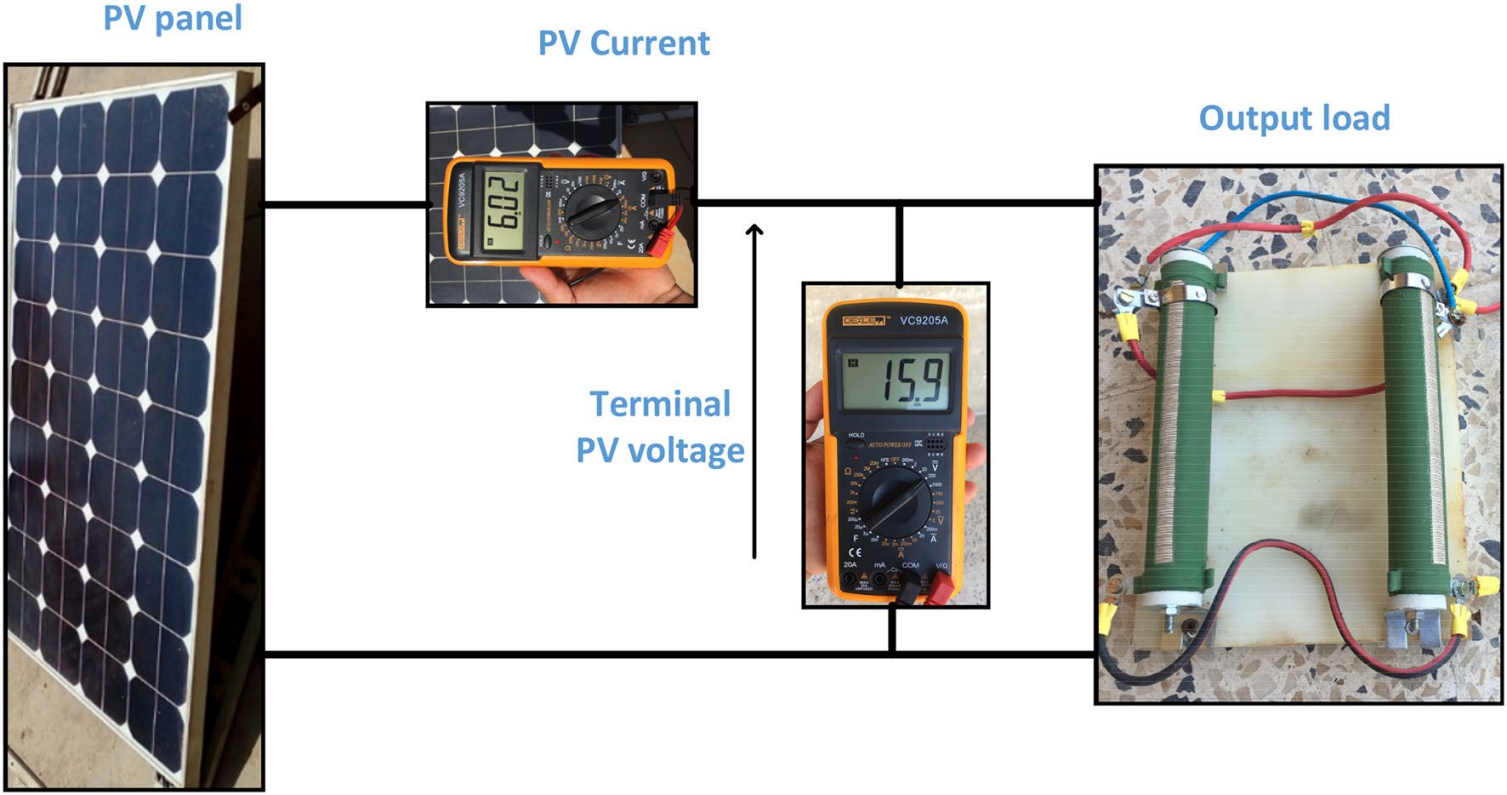
Experimental components of the proposed PV system.

**Figure 15:**
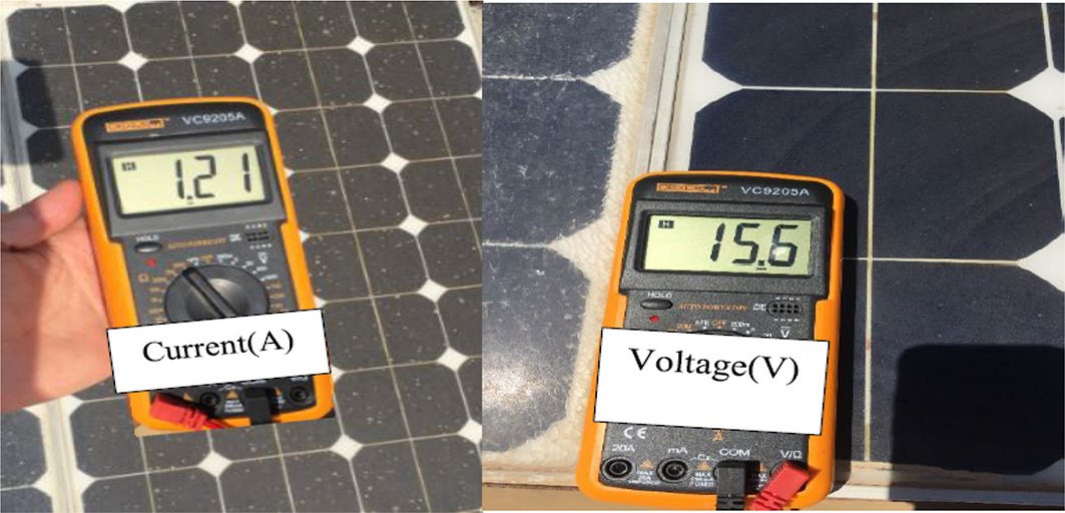
Experimental current and voltage of the proposed PV system.

**Figure 16:**
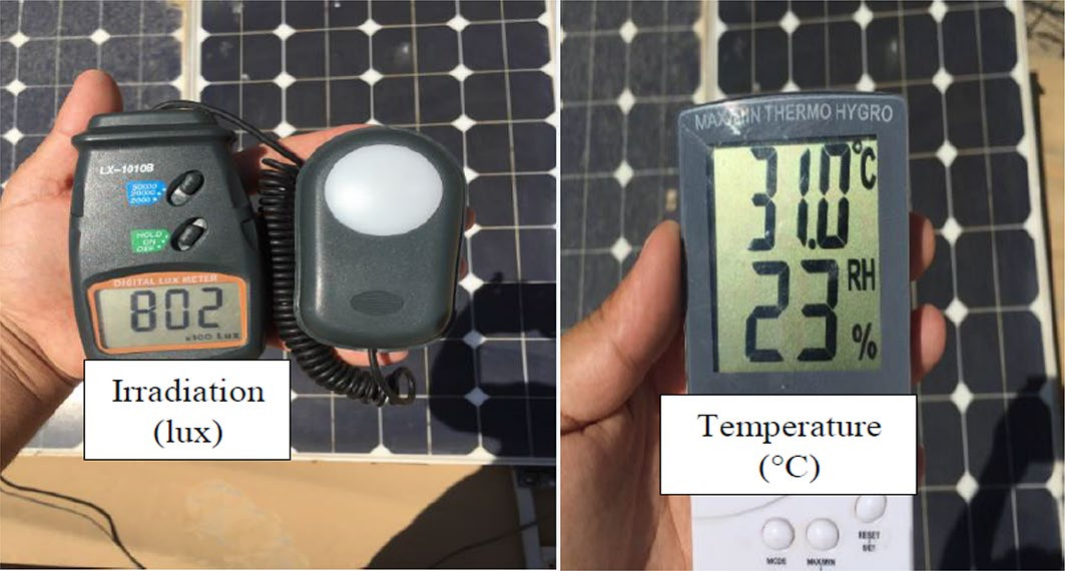
Measurements of irradiance and temperature for the proposed PV system.

**Figure 17:**
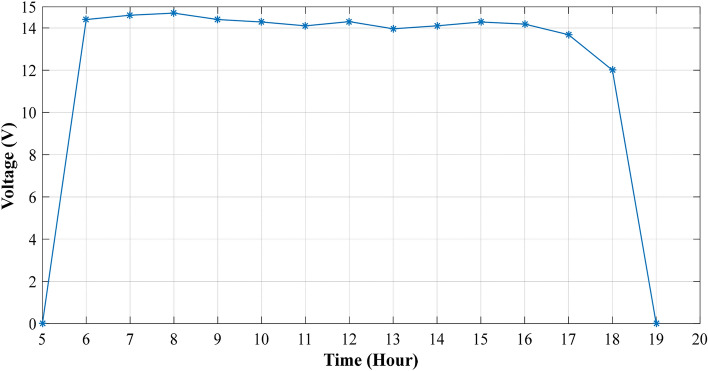
Experimental voltage of the NST-PV panel.

**Figure 18:**
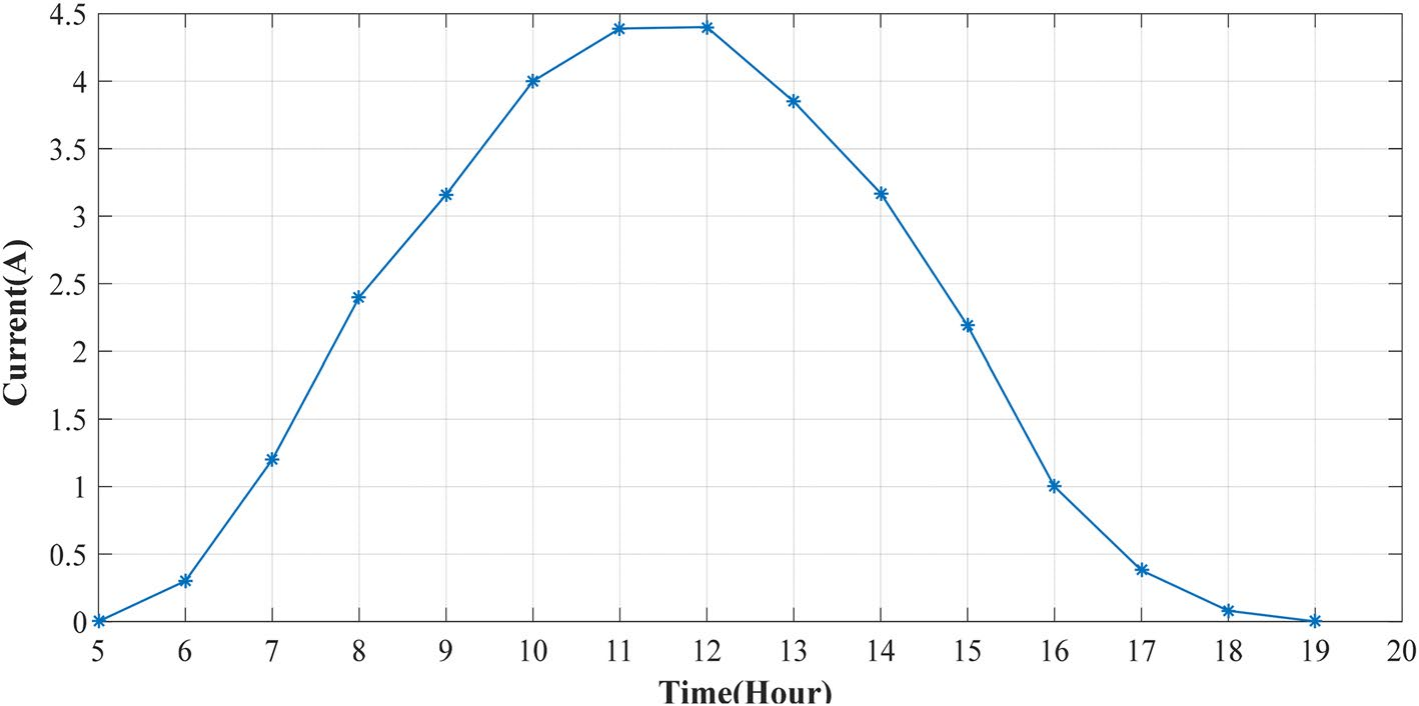
Experimental current of the NST-PV module.

**Figure 19:**
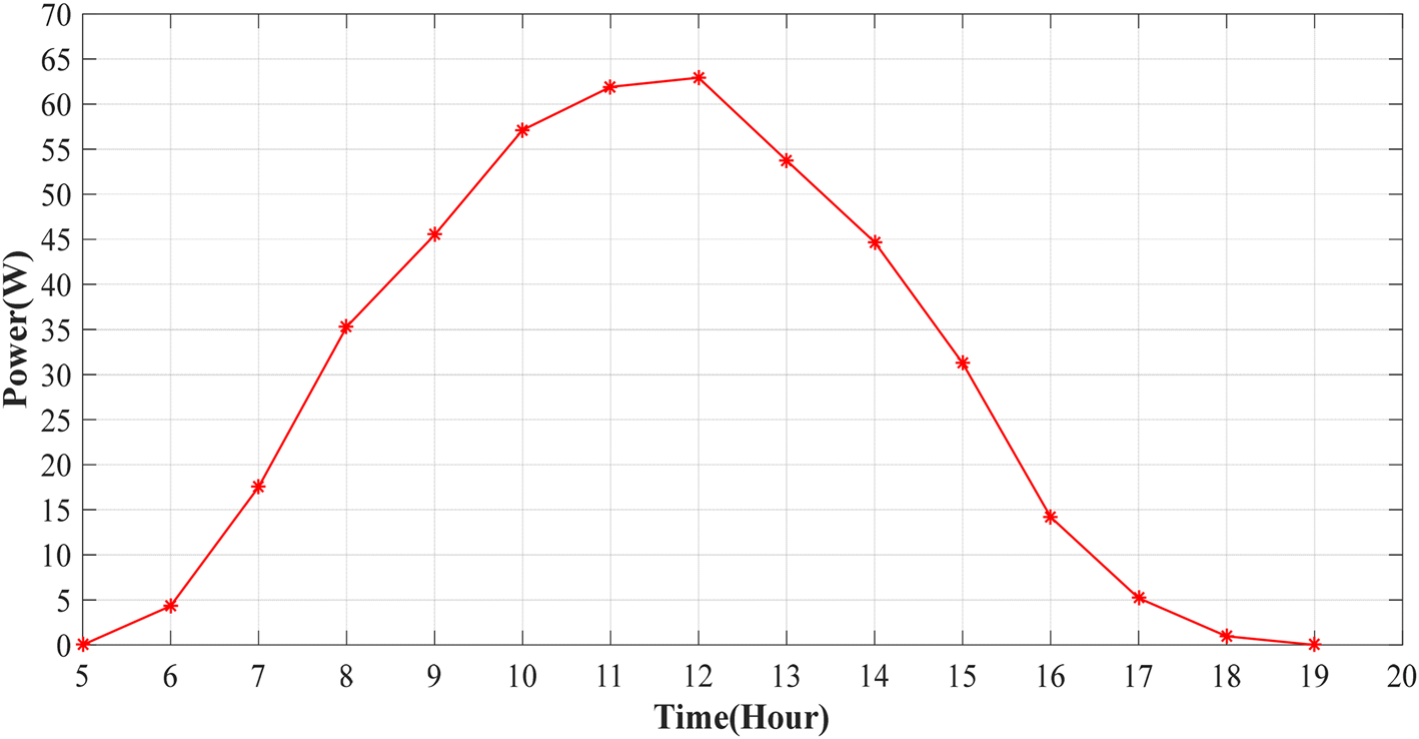
Experimental power of the NST PV module.

**Figure 20:**
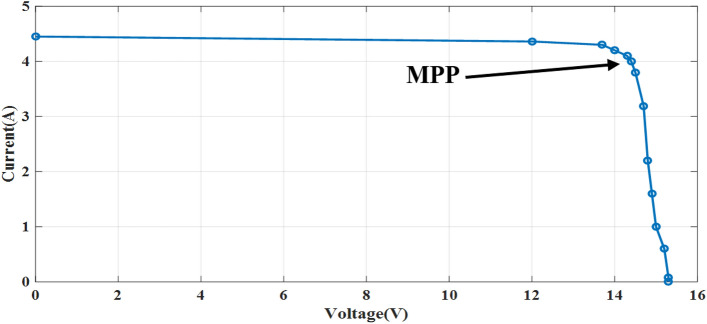
Experimental *I*–*V* graph of the NST PV module.

**Figure 21:**
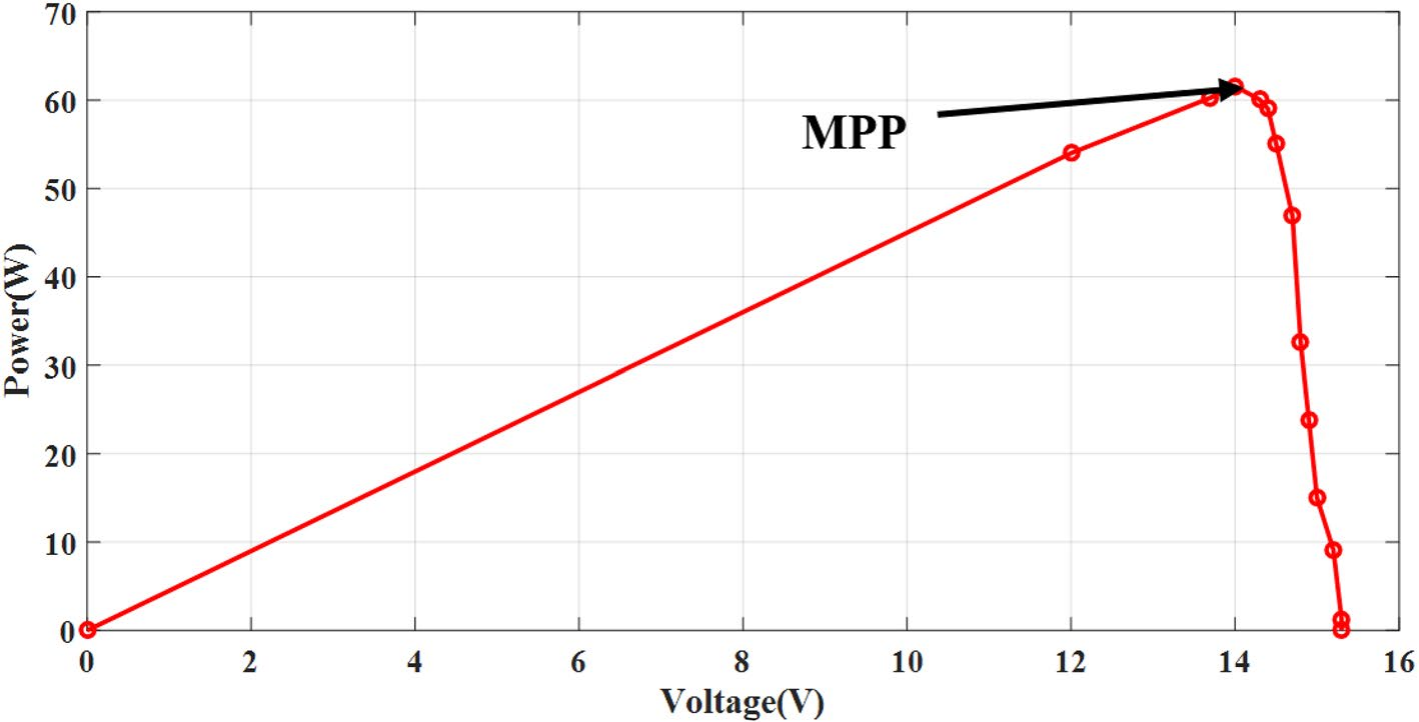
Experimental *P*–*V* graph of the NST PV module.

The experiment is conducted to validate the effectiveness of proposed method. The PV panel measurement data (voltage, current, temperature and irradiation) in one summer day for NST-120 W PV panel are obtained for different weather conditions. The experimental results are extracted through a variable load to obtain the *I*–*V* and *P*–*V* graphs. The experimental system components of the PV system are presented in Fig. 14. The photography of measurements in this experiment are shown in Figs. 15 and 16. The collected data of the real implementation are shown in Table 3. The experimental results of the voltage, current, and power of the NST-PV module for one day are reported in Figs. 17, 18, 19, respectively. The output voltage of the PV module is approximately constant due to the lower change in the ambient temperature during the experiment, except for the temperature of 20 °C in the morning. The total PV module current is mainly proportional to the irradiance and reaches to peak current at irradiance of 600 W/m^2^. Figures 20 and 21 present the *I*–*V* and *P*–*V* characteristics for one summer day, respectively, to show the NST-120 W PV module characteristics in this experiment.

**Table 3:** Experimental data for various weather conditions every 1 h for one summer day in Baghdad City, Iraq.

Time (hour)	Irradiance (W/m^2^)	Temperature (°C)	$V$ (V)	$I$ (A)	$P$ (W)
6:00 am	36	20	14.4	0.3	4.32
7:00 am	153	25	14.6	1.2	17.52
8:00 am	293	28	14.7	2.4	35.28
9:00 am	403	32	14.4	3.16	45.5
10:00 am	530	34	14.28	4	57.12
11:00 am	587	38	14.1	4.39	61.9
12:00 pm	600	40	14.3	4.4	62.92
13:00 pm	578	42	13.96	3.85	53.75
14:00 pm	490	44	14.1	3.17	44.7
15:00 pm	396	43	14.28	2.19	31.27
16:00 pm	260	43	14.18	1	14.18
17:00 pm	125	42	13.68	0.38	5.19
18:00 pm	21	40	12	0.08	0.96

## Conclusion

In this study, a simple and new method for modeling a double-diode PV model is presented. The theoretical analysis for single- and double-diode circuits is conducted. The unknown internal PV parameters are computed by using PV array tool in Simulink, and the models are modeled on the basis of their mathematical equations. The new method used in this work for modeling PV module is based on two main functions of “Multiplexer and Functions blocks” that are presented in the Simulink library. The proposed model is validated experimentally by using a monocrystalline NST-120 W PV module. The experimental results for one summer day are obtained, and the corresponding *I*–*V* and *P*–*V* characteristics are achieved accurately. The simulation and experimental results show that the double-diode model is more efficient than the single-diode model in terms of accuracy.

## Data Availability

The data that support the findings of this study are available from the corresponding author upon reasonable request.
