# Author Correction: Comparative study with practical validation of photovoltaic monocrystalline module for single and double diode models

**DOI:** 10.1038/s41598-021-01357-5

**Published:** 2021-11-02

**Authors:** Salam J. Yaqoob, Ameer L. Saleh, Saad Motahhir, Ephraim B. Agyekum, Anand Nayyar, Basit Qureshi

**Affiliations:** 1 Authority of the Popular Crowd, Baghdad, Iraq; 2 College of Engineering, Misan University, Amarah, Iraq; 3 Ecole National des sciences appliquées, Université Sidi Med Ben Abdellah, Fes, Morocco; 4 Department of Nuclear and Renewable Energy, Ural Federal University, 19 Mira Street, Ekaterinburg, Russia; 5 Graduate School, Duy Tan University, Da Nang, Vietnam; 6 College of Computer & Info Sc., Prince Sultan University, Riyadh, Saudi Arabia

Correction to: *Scientific Reports* https://doi.org/10.1038/s41598-021-98593-6, published online 27 September 2021

The original version of this Article contained an error in the Acknowledgments section.

“This research was part of solar PV system project that received funding from the Middle Technical University for Research and Innovation Program 2020, Iraq.”

now reads:

“This work was supported in part by the Robotics and Internet of Things Laboratory, Prince Sultan University, Riyadh, Saudi Arabia."

The original Article has been corrected.

